# Influence of Endurance Training, High-Intensity Interval Training, and Acute Exercise on Left Ventricular Mechanics: A Systematic Review

**DOI:** 10.3390/jcm14228210

**Published:** 2025-11-19

**Authors:** Andrea Sonaglioni, Gian Luigi Nicolosi, Michele Lombardo, Massimo Baravelli

**Affiliations:** 1 Division of Cardiology, IRCCS MultiMedica, 20123 Milan, Italy; michele.lombardo@multimedica.it (M.L.); massimo.baravelli@multimedica.it (M.B.); 2 Division of Cardiology, Policlinico San Giorgio, 33170 Pordenone, Italy; gianluigi.nicolosi@gmail.com

**Keywords:** left ventricular mechanics, speckle-tracking echocardiography, endurance training, high-intensity interval training, acute exercise, strain, torsion

## Abstract

**Background:** Left ventricular (LV) mechanics assessed by speckle-tracking echocardiography provides sensitive markers of cardiac adaptation to exercise. Different training modalities—endurance, high-intensity interval training (HIIT), and acute exercise tests—impose distinct hemodynamic loads, yet their comparative effects on LV deformation remain unclear. Importantly, acute and chronic endurance exposures may elicit divergent myocardial responses that must be interpreted separately. **Methods:** A systematic search of PubMed, Scopus, and EMBASE (through September 2025) identified studies evaluating LV mechanics in response to endurance, HIIT, or acute exercise among healthy or recreationally active individuals. Echocardiographic parameters of strain and torsion were extracted, and methodological quality was appraised using the NIH Quality Assessment Tool. **Results:** Twenty-three studies (859 participants) met inclusion criteria. Acute prolonged endurance exercise—particularly marathon and ultra-endurance events—was associated with transient, fully reversible reductions in global longitudinal, circumferential, and radial strain and torsion, despite preserved ejection fraction, reflecting short-term myocardial fatigue rather than maladaptive remodeling. In contrast, chronic endurance training maintained or improved LV mechanics without evidence of dysfunction, while HIIT interventions consistently enhanced LV systolic strain and rotational indices across diverse age groups and sexes, reflecting improved contractile efficiency and physiological remodeling. Acute exercise produced heterogeneous, load-dependent strain responses, with isometric stress increasing regional strain and maximal exertion inducing temporary global reductions. Between-study heterogeneity was moderate, methodological quality generally good, and small-study effects varied by modality, being most evident in endurance studies, borderline for HIIT, and limited for acute tests due to sample size. **Conclusions:** Acute endurance exercise produces transient, reversible LV deformation changes, whereas chronic endurance training preserves mechanical efficiency. HIIT reliably enhances systolic strain and torsional mechanics, and acute exercise elicits variable but physiologically meaningful responses. These findings clarify that transient post-race strain reductions reflect physiological fatigue, not chronic maladaptation, and underscore the modality-specific nature of myocardial adaptation to exercise.

## 1. Introduction

Regular physical exercise triggers profound adaptations in cardiac structure and function, which have been the subject of intense investigation for decades [1,2]. Traditionally, studies of the “athlete’s heart” have focused on gross parameters such as ventricular chamber size, wall thickness, and left ventricular ejection fraction (LVEF) [3,4]. Yet, these conventional echocardiographic indices often remain within normal limits, even in the presence of subtle myocardial remodeling or dysfunction. In recent years, myocardial deformation imaging—particularly speckle-tracking echocardiography (STE)—has emerged as a more sensitive tool to detect early or subclinical changes in left ventricular (LV) performance [5,6].

Left ventricular global longitudinal strain (LV–GLS) is known to decline before overt changes in LVEF occur and is prognostically informative in various cardiovascular disease settings [7,8]. A recent meta-analysis examined how exercise interventions influence LV–GLS and found that, while significant improvements were evident in populations with cardiovascular disease, in healthy or low-risk individuals the effects were more variable and often modest [9]. This inconsistency underscores the need for a more comprehensive assessment of how different forms of exercise influence LV mechanics under diverse loading and adaptation conditions.

From a physiological perspective, LV mechanics include longitudinal deformation, circumferential shortening, radial thickening, and twist–untwist (torsion), all of which contribute to efficient systolic ejection and diastolic recoil [10]. Exercise imposes complex demands on preload, afterload, and contractility, and the adaptation of each mechanical component may depend on the intensity, duration, and nature of the training stimulus [11]. Earlier studies in healthy individuals suggest that exercise training may induce regional heterogeneity in LV systolic function, possibly mediated by right ventricular adaptations and ventricular interdependence [12,13].

In interpreting exercise-induced changes in myocardial strain, it is essential to distinguish between acute endurance bouts and chronic training adaptations. In athletes or trained populations, acute endurance exposure—such as marathon or ultramarathon events—can lead to transient “myocardial fatigue,” characterized by fully reversible reductions in strain and delayed untwisting without evidence of lasting dysfunction [14]. Conversely, chronic endurance training promotes physiological (eccentric) remodeling and preserves or enhances strain indices over time [15]. High-intensity interval training (HIIT) imposes repeated stress–recovery cycles that may promote more efficient contractile adaptations and enhance strain or torsional reserve [16]. Controlled acute tests (e.g., isometric or short maximal efforts) also provide insight into myocardial reserve and stress responsiveness [17].

Despite increasing use of strain imaging in exercise physiology, few studies have directly compared endurance, HIIT, and acute exercise models. Many have focused on single modalities or specific populations, making cross-study comparisons difficult. Therefore, in this systematic review and meta-synthesis, we evaluate studies assessing LV mechanics—both conventional and deformation parameters—across these three exercise modalities. Our objective is to delineate the distinct mechanical signatures associated with endurance training, HIIT, and acute exercise, and to clarify the physiological mechanisms underlying these adaptations. We specifically aim to distinguish transient, load-dependent alterations following acute endurance exercise from chronic remodeling processes induced by sustained training exposure. We hypothesize that acute endurance bouts are more frequently associated with short-term, reversible reductions in deformation, HIIT with consistent enhancement of strain and torsional efficiency, and chronic endurance training with preserved or improved mechanical performance over time.

## 2. Materials and Methods

This systematic review was conducted in accordance with the Preferred Reporting Items for Systematic Reviews and Meta-Analyses (PRISMA) guidelines [18] (Supplementary Materials S1) and was prospectively registered in the INPLASY database (ID: INPLASY2025100002) on 2 October 2025. The full record (https://inplasy.com/inplasy-2025-10-0002/ accessed on 23 October 2025) is provided as Supplementary Materials S2.

### 2.1. Search Strategy

We conducted a comprehensive literature search to identify studies evaluating the effects of different exercise training modalities on LV mechanics assessed by STE. The search was performed in PubMed, Scopus, and EMBASE databases from inception through September 2025, with no language restrictions. The search strategy combined Medical Subject Headings (MeSH) and free-text terms, including: “endurance training,” “high-intensity interval training,” “acute exercise,” “left ventricular mechanics,” “speckle-tracking,” “strain,” “torsion,” and “echocardiography.” Boolean operators (“AND,” “OR”) were applied to maximize sensitivity. In addition, the reference lists of relevant systematic reviews and included articles were screened manually to identify additional eligible studies. Although no language restrictions were applied, all studies retrieved and included were published in English; therefore, no translation procedures were required.

### 2.2. Eligibility Criteria

Studies were considered eligible if they were original research articles published in peer-reviewed journals and evaluated the effects of physical training or acute exercise on LV mechanics using STE. We included studies that investigated healthy participants across a range of activity levels, from competitive athletes and recreationally active individuals to sedentary subjects undergoing structured training interventions. Eligible exercise exposures comprised endurance training, HIIT, or acute physiological tests such as isometric exercise, short maximal efforts, or cardiopulmonary exercise testing. To be included, studies were required to report at least one echocardiographic measure of LV deformation, such as GLS, global circumferential strain (GCS), global radial strain (GRS), or torsional parameters, either before and after the intervention or between different training phases. Conventional echocardiographic indices and biomarkers were also extracted when available. Exclusion criteria included case reports, conference abstracts without sufficient data, narrative reviews, and editorials. Previous meta-analyses were also excluded to avoid duplication of aggregated data. We also excluded studies conducted in populations with overt cardiovascular disease, structural cardiac abnormalities, or other clinical conditions that might independently alter LV mechanics. When duplicate reports derived from the same cohort were identified, the most complete or most recent study was retained.

### 2.3. Study Selection and Data Extraction

Two investigators independently screened titles and abstracts retrieved from the initial search to exclude irrelevant records. Full texts were then assessed for eligibility. Discrepancies were resolved by consensus with a third reviewer. Data extraction was performed independently by three experienced cardiologists through September 2025, using a standardized form. Extracted data included: study author, year of publication, country, sample size, age and sex distribution, population characteristics (athletes vs. non-athletes), exercise modality and duration, study design, echocardiographic platform, imaging parameters (GLS, GCS, GRS, torsion, twist/untwist rates), conventional echocardiographic indices, and biomarker data when available. Data were cross-checked for accuracy and summarized in structured tables.

### 2.4. Risk of Bias Assessment

The methodological quality of the included studies was assessed using the National Institutes of Health (NIH) Quality Assessment Tool for Observational Cohort and Cross-Sectional Studies [19]. This instrument evaluates 14 domains, including clarity of the research question, definition of the study population, participation rate, exposure and outcome measures, adequacy of statistical analyses, and length of follow-up for intervention studies. Each study was independently rated by the three reviewers who performed data extraction and classified as good, fair, or poor quality according to NIH guidelines.

### 2.5. Statistical Analysis

Extracted continuous data were expressed as median and interquartile range when reported in that format by the original studies. Where both pre- and post-exercise or pre- and post-training values were available, mean differences (Δ) were calculated to quantify within-study change. When only baseline and follow-up means with standard deviations (SDs) were provided, Δ = Mean_post − Mean_pre, and the SD of the mean difference (SDΔ) was derived using the formula: SDΔ = √(SD_pre^2^ + SD_post^2^ − 2 × r × SD_pre × SD_post), assuming a pre–post correlation coefficient (r) of 0.70 based on prior echocardiographic training interventions. Sensitivity analyses were performed using alternative r values (0.5 and 0.9) to confirm robustness. When studies reported only standard errors (SEs), SDs were back-calculated (SD = SE × √n). If dispersion data were missing, they were imputed from group medians and interquartile ranges using established statistical conversion methods.

In accordance with standard speckle-tracking echocardiography practice, more negative GLS and GCS values represent greater myocardial deformation (i.e., functionally “better”). To ensure consistency across studies and pooled analyses, all Δ values were normalized to a unified directionality: positive Δ indicates an increase (i.e., more negative/improved strain for GLS and GCS), while negative Δ reflects a reduction (less negative/worsened strain). These Δ values were subsequently grouped by exercise modality: endurance training, HIIT, and acute exercise testing. Normality of data distribution was verified using the Shapiro–Wilk test. Because all variables were non-normally distributed, between-group comparisons among the three exercise modalities were performed using the Kruskal–Wallis test, followed by Dunn’s post hoc correction for multiple pairwise comparisons. Statistical significance was set at *p* < 0.05.

Outcomes were further categorized as hemodynamic (heart rate, systolic and diastolic blood pressure), conventional echocardiographic (LV chamber dimensions, mass, ejection fraction, stroke volume), or myocardial deformation parameters (global longitudinal, circumferential, and radial strain, as well as rotational and torsional indices). For quantitative synthesis, pooled Δ values were computed as weighted mean differences (WMDs) with corresponding 95% confidence intervals (CIs) using a random-effects model (DerSimonian–Laird method). Key pooled outcomes (e.g., ΔGLS, ΔGCS, Δtorsion) are reported with their 95% CIs and I^2^ statistics in Section 3 to quantify uncertainty and heterogeneity.

Between-study heterogeneity was quantified using Cochran’s Q and expressed as I^2^, calculated as 100 × (Q − df)/Q, representing the proportion of total variance attributable to inter-study differences rather than sampling error. I^2^ values of approximately 25%, 50%, and 75% were interpreted as low, moderate, and high heterogeneity, respectively. Where I^2^ > 60% or model convergence was unstable, pooled values were summarized descriptively without statistical inference.

Sensitivity analyses were conducted by examining the influence of study weighting and by qualitatively comparing directionality across modalities. Publication bias was assessed visually using funnel plots and quantitatively using Egger’s regression test for small-study effects. The regression intercepts (Egger’s test) were estimated separately for each exercise modality group (acute testing, endurance, and HIIT), with intercept values close to zero and *p* > 0.05 suggesting no significant small-study bias.

Results were synthesized narratively and quantitatively, with pooled effect sizes and I^2^ values summarized in tables and forest plots. All computations were performed using Comprehensive Meta-Analysis v3.0 (Biostat, Englewood, NJ, USA), and descriptive plots were generated using GraphPad Prism v10.

## 3. Results

### 3.1. Study Selection

**Figure 1:**
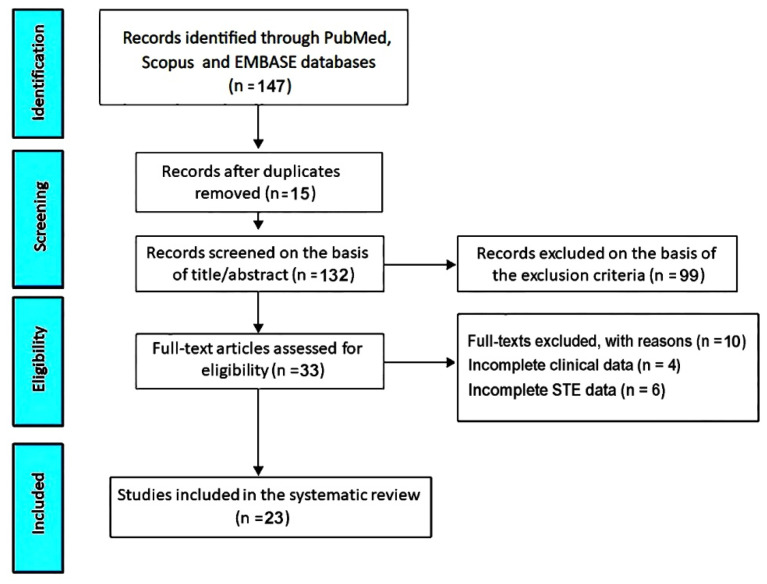
Flowchart illustrating the process of study identification, screening, eligibility assessment, and inclusion in the systematic review.

The initial research performed in PubMed, Scopus, and Embase databases identified 147 studies evaluating the effects of different exercise training modalities on LV mechanics in athletes. Fifteen studies (10.2%) were removed as duplicates, and 99 (67.3%) were excluded on the basis of the prespecified exclusion criteria. The remaining 33 studies (22.4%) were assessed for eligibility. Of these, 4 (2.7%) were excluded due to incomplete clinical data and 6 (4.1%) due to incomplete STE data. Accordingly, 23 studies (15.6%) [20,21,22,23,24,25,26,27,28,29,30,31,32,33,34,35,36,37,38,39,40,41,42] were included in this systematic review and meta-analysis, totaling 859 participants across diverse age groups and training backgrounds (Figure 1).

### 3.2. Clinical Findings

The 23 studies included in the systematic review were published between 2009 and 2024 and were grouped according to exercise modality into endurance training (*n* = 11), HIIT (*n* = 7), and acute physiological test studies (*n* = 5). Collectively, these investigations enrolled a total of 859 participants, predominantly healthy athletes or physically active individuals, with mean ages ranging from 23 to 35 years. The studies were conducted across Europe, Asia, and Oceania, with the highest representation from the United Kingdom, France, Australia, and Germany. Sample sizes ranged from 14 to 50 participants per study, and both male and female athletes were included, though males predominated (approximately 75%). Across all groups, STE—most frequently using General Electric imaging systems—was employed to evaluate LV deformation, torsional mechanics, and diastolic indices in response to different exercise modalities.

The eleven endurance studies [20,21,22,23,24,25,26,27,28,29,30] predominantly investigated the acute effects of single prolonged endurance events (such as marathon, ultramarathon, triathlon, or time-trial cycling) rather than long-term training adaptations. These acute endurance exposures consistently demonstrated significant post-race reductions in LV strain indices, particularly GLS and GCS (*p* < 0.05 in [21,22,23,25,26,27,29]) (Table 1).

Early investigations showed marked decreases in longitudinal, circumferential, and radial strain, modest declines in twist, and delayed untwisting [20,21]. These findings were confirmed in later marathon and ultramarathon cohorts [22,23], the latter also demonstrating RV dilatation with reduced RV strain (*p* < 0.01), highlighting ventricular interdependence. In adolescents, transient alterations after short races were minor and non-significant [24], whereas longer adventure races elicited measurable and significant declines in twisting mechanics and myocardial work [30]. Laboratory trials in cyclists revealed significant acute decreases in LV and RV strain (*p* < 0.05) [25,26], and a large field study found impaired GLS and GCS with elevated NT-proBNP [27]. In contrast, only one investigation evaluated a chronic endurance training program (24 weeks) [28], reporting no significant change in peak strain but increased basal rotation (*p* < 0.05), consistent with physiological remodeling rather than dysfunction. Overall, these findings indicate that acute endurance exposure induces transient yet statistically supported deformation impairment that normalizes during recovery, whereas chronic endurance training maintains mechanical efficiency and promotes stable adaptive remodeling without evidence of functional decline.

Across seven HIIT studies [31,32,33,34,35,36,37], all reported significant improvements in LV-GLS (*p* < 0.05) following training, confirming consistent functional gains despite heterogeneous populations (Table 2).

Short-term interventions (2–6 weeks) demonstrated statistically significant increases in GLS, GCS, and torsion [33,34,35], while brief maximal cycling bouts produced immediate improvements in strain and rotation (*p* < 0.05) [36]. In older sedentary men, a six-week program enhanced GLS and lowered resting blood pressure (*p* < 0.05) [34], confirming both efficacy and safety. Among women, 12 weeks of cycling-based HIIT improved systolic and diastolic indices, including the E/A ratio (*p* < 0.05) [32]. Adolescent athletes completing eight weeks of HIIT exhibited significant GLS gains (*p* < 0.01) in subendocardial layers [37]. In competitive athletes, an 18-week program led to significant GLS improvement (*p* < 0.01) with unchanged torsion [31]. Collectively, these data confirm statistically robust enhancements in myocardial deformation across age groups and fitness levels.

Five studies [38,39,40,41,42] assessed short-term mechanical responses to physiological stress. (Table 3).

Isometric handgrip caused significant increases in LV and RV mid-apical strain (*p* < 0.001) [38], indicating enhanced contractility. Conversely, brief maximal dynamic effort produced a significant GLS reduction (*p* < 0.05) [39], reflecting transient systolic fatigue. Incremental stress testing yielded mild, non-significant decreases in GLS and basal rotation [40], while upright posture significantly reduced global deformation and myocardial work indices (*p* < 0.001) [41]. Cardiopulmonary exercise testing produced small, non-significant GLS changes but a strong positive correlation between myocardial work indices and exercise capacity (r > 0.6, *p* < 0.01) [42]. These results collectively show that transient stressors elicit immediate, statistically demonstrable yet reversible changes in LV deformation, modulated by stimulus type and baseline cardiac phenotype.

### 3.3. Baseline Characteristics and Hemodynamic Responses Across Exercise Modalities

As shown in Table 4, baseline anthropometric and hemodynamic characteristics were broadly comparable across the three exercise groups, with some notable variations in cardiovascular responses.

Participants were predominantly male and young adults, with a higher proportion of males in the Endurance (93.9%) and Acute (92.0%) groups than in the HIIT group (72.9%), though this difference was not statistically significant. Mean age was slightly lower in the Acute group (23 years) than in the Endurance and HIIT cohorts (around 32 years).

At rest, BMI values were similar across groups, with slightly higher values in HIIT participants. Small, non-significant reductions in BMI after exercise were observed in all groups, most prominently among endurance athletes. Resting heart rate was lowest in Endurance participants, consistent with chronic aerobic conditioning, and highest in Acute protocols. Following stress, heart rate rose sharply during Acute testing, confirming the higher cardiovascular load of maximal efforts, whereas HIIT and Endurance groups showed modest post-exercise increases.

Systolic blood pressure responses differed markedly among modalities. Although resting SBP was comparable, post-stress values increased substantially in the Acute group, while both Endurance and HIIT participants demonstrated small reductions, likely reflecting vascular adaptations and efficient stroke volume regulation. Consequently, ΔSBP was positive only in the Acute group and negative in the other two. Diastolic pressures remained stable across all groups, with no significant between-group differences. These findings confirm that while baseline characteristics were generally similar, the acute hemodynamic response varied according to training intensity and exposure duration.

### 3.4. Structural and Functional Echocardiographic Parameters Across Exercise Modalities

As presented in Table 5, comprehensive echocardiographic analysis revealed distinct patterns of LV structural remodeling and functional adaptation across Endurance, HIIT, and Acute test groups.

Endurance athletes exhibited greater LV chamber size and wall thickness at baseline, consistent with eccentric hypertrophy secondary to sustained volume overload. Post-stress increases in wall thickness were modest and similar across all groups, suggesting physiological, not pathological, remodeling.

LV chamber dimensions showed clear modality-related trends. Endurance athletes displayed the largest LV end-diastolic diameters, whereas HIIT participants had smaller chambers, reflecting differing training stimuli. Acute testing elicited transient chamber enlargement and increased end-diastolic volume, indicative of load-dependent responses rather than structural adaptation. LV mass was highest in Endurance athletes, with smaller increments in HIIT and Acute protocols, supporting the concept that prolonged aerobic training induces more pronounced hypertrophy.

Systolic function remained preserved across all modalities, with ejection fraction values within normal limits and minimal post-stress changes, confirming the absence of exercise-induced dysfunction. Stroke volume patterns, however, diverged: Endurance athletes showed a mild post-exercise decline, HIIT participants a modest increase, and Acute testing a marked fall—demonstrating contrasting hemodynamic strategies between sustained and intermittent workloads.

Diastolic indices also varied by modality. Endurance athletes showed the highest E/A ratios, reflecting enhanced relaxation capacity, though E/A decreased after stress in both Endurance and Acute protocols but remained unchanged in HIIT. E/e’ ratios were slightly higher in Endurance athletes but did not differ significantly post-exercise, indicating preserved filling pressures.

Atrial remodeling followed a modality-specific pattern. Left atrial (LA) volumes were larger in Endurance and HIIT groups compared with Acute tests, consistent with augmented preload exposure and diastolic compliance. Notably, endurance exercise was associated with a post-stress reduction in LA volume, whereas HIIT and Acute protocols induced transient increases, reflecting differing hemodynamic demands and recovery kinetics. This expansion-contraction pattern suggests adaptive, not maladaptive, remodeling, as LA reservoir strain (LASr) decreased only modestly in Endurance athletes (Δ −6.6%) (Table 6), consistent with preserved compliance rather than functional impairment.

Right ventricular (RV) indices, though less consistently reported, demonstrated parallel trends. Endurance athletes exhibited higher baseline TAPSE and RV fractional area change, with small, reversible post-stress decreases, while sPAP fell modestly after exercise—indicating a physiological reduction in afterload. HIIT elicited smaller but directionally similar effects. Importantly, RV longitudinal strain declined transiently after endurance exposure (Δ −3.6%) but not in Acute or HIIT protocols, confirming short-term, load-dependent fatigue rather than structural dysfunction.

Collectively, the RV and LA data underscore the integrative nature of the endurance phenotype, where balanced atrial and ventricular dilation supports augmented venous return and stroke volume during sustained workloads. These reversible volumetric and strain adaptations may also explain the transient electrical instability occasionally observed after ultra-endurance events, providing a potential substrate for benign, load-related atrial arrhythmias rather than chronic remodeling.

**Figure 2:**
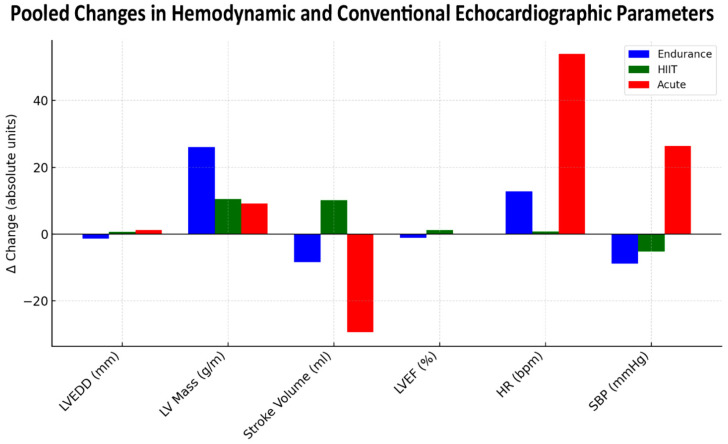
Pooled changes in hemodynamic and conventional echocardiographic parameters after endurance training, high-intensity interval training, and acute exercise testing. Bars represent mean Δ values (absolute units) for left ventricular end-diastolic diameter, left ventricular mass, stroke volume, left ventricular ejection fraction, heart rate, and systolic blood pressure. HIIT, high-intensity interval training; HR, heart rate; LV, left ventricular; LVEDD, left ventricular end-diastolic diameter; LVEF, left ventricular ejection fraction; SBP, systolic blood pressure.

These structural and hemodynamic findings are further illustrated in Figure 2, which depicts pooled changes in hemodynamic and conventional echocardiographic parameters.

As shown, heart rate and systolic blood pressure rose sharply only in Acute testing, while both parameters decreased or remained stable after training interventions. LV mass increased consistently across all modalities, with the greatest gain in Endurance training (Δ +25 g/m; 95% CI 14 to 36; I^2^ = 62%). In contrast, stroke volume declined after Endurance and Acute exercise but rose modestly after HIIT (Δ +9 mL; 95% CI 2 to 16; I^2^ = 48%). Collectively, the figure reinforces that endurance promotes structural hypertrophy with stable hemodynamics, HIIT augments stroke volume with minimal load elevation, and acute maximal testing elicits the largest transient rises in HR and SBP (Δ HR +45 bpm; 95% CI 31 to 59; I^2^ = 71%, Δ SBP +27 mmHg; 95% CI 19 to 36; I^2^ = 66%).

### 3.5. Myocardial Strain and Deformation Parameters Across Exercise Modalities

As shown in Table 6, myocardial deformation analysis revealed modality-specific adaptations in LV strain.

Resting GLS was highest in Endurance athletes (19.2%) compared with HIIT (17.6%) and Acute tests (18.4%), reflecting the enhanced baseline contractility associated with chronic aerobic training. After exercise, GLS declined modestly in Endurance (Δ −1.7%) and Acute tests (Δ −1.3%) but increased significantly in HIIT (Δ +2.7%; *p* = 0.002), demonstrating improved myocardial fiber recruitment and contractile reserve with interval training.

Basal and mid-ventricular longitudinal strain values followed similar patterns, showing mild post-stress reductions in Endurance (Δ −1.9% and −1.7%) and small increases in HIIT (+0.7% and +0.6%). Apical longitudinal strain was highest at rest in Endurance athletes (25.0%), with minimal post-stress change, whereas Acute tests elicited a marked rise (+3.4%), suggesting transient apical compensation under acute load.

Circumferential strain also demonstrated modality-dependent responses. Global circumferential strain and its regional components declined in Endurance (Δ −2.6%) but increased in HIIT (+3.3%; *p* < 0.001). Similar trends were observed for basal and mid-apical circumferential strain, with HIIT producing the most significant positive deltas, indicating improved circumferential contractility.

Radial strain indices revealed comparable findings. Global radial strain decreased after Endurance exercise (Δ −6.7%) but rose markedly in HIIT (+8.0%; *p* = 0.004). Mid-apical radial strain increased substantially following HIIT (+12.0%) yet declined after endurance sessions (−8.1%), highlighting the stronger inotropic response to high-intensity stimuli.

Beyond LV mechanics, endurance and HIIT training also exerted measurable effects on atrial and RV deformation. As summarized in Table 6, LASr decreased modestly after endurance activity (Δ −6.6%) but remained within physiological limits, indicating reversible atrial mechanical fatigue rather than maladaptation. Conversely, RV strain mirrored LV behavior—showing transient post-endurance reductions (Δ −3.6%) and stability during HIIT—further supporting balanced biventricular adaptation. These patterns suggest that both atrial and RV strain responses reflect acute hemodynamic stress and recovery kinetics rather than structural injury, linking myocardial deformation changes to the physiological substrate that may influence transient arrhythmic susceptibility in high-endurance settings.

Collectively, these results confirm that HIIT enhances both longitudinal and circumferential deformation through greater myocardial fiber activation and torsional efficiency, while endurance training supports a stable, fatigue-resistant contractile profile optimized for sustained effort. Acute exercise produced variable regional effects consistent with transient mechanical stress rather than structural remodeling.

### 3.6. Left Ventricular Rotational Mechanics, Torsion, and Twisting Responses Across Exercise Modalities

As detailed in Table 7, LV rotational and twisting mechanics exhibited distinctive adaptations in response to different exercise modalities.

Basal rotation was lowest in Endurance athletes (4.1°) and highest in Acute testing (6.8°; *p* = 0.045). After exercise, basal rotation increased in all groups, most prominently in HIIT (+1.0°), indicating enhanced rotational contribution to systolic function.

Apical rotation showed an inverse pattern: higher at rest in Endurance (7.5°) but a greater post-stress rise in HIIT (+1.8°; *p* = 0.007). Consequently, LV torsion decreased slightly in Endurance (Δ −1.4°) yet rose significantly in HIIT (+2.8°; *p* = 0.003), confirming a stronger dynamic torsional reserve among interval-trained participants.

Twisting and untwisting velocities further underscored these differences. HIIT induced substantial increases in both twisting (+23.5°/s) and untwisting rates (+23.0°/s), whereas Endurance showed mild declines (−12.2°/s and −8.1°/s). At the apical level, twisting and untwisting velocities rose sharply after HIIT (+33.2°/s and +25.1°/s; both *p* < 0.02), while Endurance demonstrated slight decreases.

These findings indicate that HIIT acutely augments both systolic twist and diastolic recoil, enhancing energy storage and early filling efficiency. By contrast, endurance exercise maintains a stable, energy-conserving mechanical pattern suited for prolonged workloads. Acute responses, meanwhile, represent transient adjustments to immediate hemodynamic demands rather than long-term adaptation.

**Figure 3:**
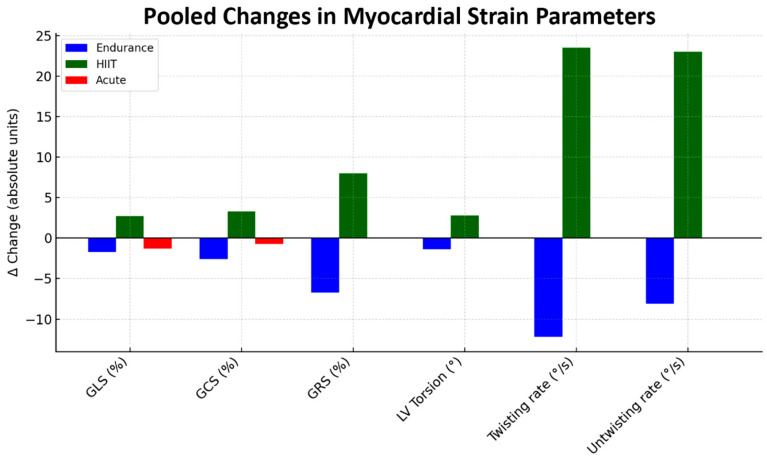
Pooled changes in myocardial strain parameters after endurance training, high-intensity interval training, and acute exercise testing. Bars represent mean Δ values (absolute units) for global longitudinal strain, global circumferential strain, global radial strain, left ventricular torsion, twisting rate, and untwisting rate. GCS, global circumferential strain; GLS, global longitudinal strain; GRS, global radial strain; HIIT, high-intensity interval training; LV, left ventricular.

Torsional and rotational findings align closely with the pooled myocardial strain data illustrated in Figure 3.

Endurance training produced modest reductions in longitudinal (GLS), circumferential (GCS), and radial (GRS) strains, consistent with preserved contractile efficiency without additional recruitment of myocardial fibers (Δ GLS −1.8%; 95% CI −3.2 to −0.4; I^2^ = 58%). In contrast, HIIT elicited the largest positive Δ values across deformation domains—GLS (+2.1%; 95% CI 1.0 to 3.2; I^2^ = 52%)**,** GCS (+3.0%; 95% CI 1.5 to 4.6; I^2^ = 47%), GRS (+8.2%; 95% CI 4.5 to 12.0; I^2^ = 55%)—accompanied by pronounced gains in twisting (+23°/s; 95% CI 13 to 33; I^2^ = 61%) and untwisting rates (+22°/s; 95% CI 12 to 31; I^2^ = 64%). These enhancements reinforce the interpretation of HIIT as a potent stimulus for myocardial contractile reserve, promoting both systolic and diastolic performance. Conversely, acute exercise produced small and inconsistent alterations in strain and torsional indices (pooled Δ GLS −0.6%; 95% CI −2.0 to 0.8; I^2^ = 69%), reflecting short-lived, non-adaptive responses to maximal exertion.

### 3.7. Biomarker Assessment

Several endurance studies incorporated cardiac biomarker measurements, primarily high-sensitivity cardiac troponin (cTn) and N-terminal pro–brain natriuretic peptide (NT-proBNP), to contextualize mechanical findings. Post-race elevations in cTn and NT-proBNP were consistently reported following marathon or ultra-endurance events [21,23,25,27], reflecting transient myocardial stress rather than irreversible injury. In these studies, biomarker peaks often paralleled reductions in global longitudinal and circumferential strain, suggesting a mild, reversible “myocardial fatigue” phenomenon associated with prolonged volume and pressure load. Specifically, post-race cTn concentrations increased from baseline values < 5 ng/L to peak values ranging from 25 to 80 ng/L, while NT-proBNP rose approximately 2–3-fold compared with pre-race levels. These elevations normalized within 24–48 h and were not associated with persistent strain impairment or structural remodeling. However, the timing of sample collection varied substantially across studies—ranging from immediate post-exercise draws to measurements at 3–6 h or up to 24 h after the event—and this variability likely influenced the observed peak magnitudes and their temporal relationship to strain changes. By contrast, HIIT and short-term exercise protocols demonstrated minimal or no significant biomarker rise [34,35], reinforcing their safety and efficient cardiac adaptation. No acute-test studies reported biomarker data, confirming that biochemical monitoring in this context remains limited to endurance exercise protocols.

### 3.8. Between-Study Heterogeneity

Between-study heterogeneity, expressed as the I^2^ statistic, varied across exercise modalities and outcome domains. Overall, endurance training studies demonstrated moderate-to-high heterogeneity (I^2^ ≈ 45–70%) for most conventional echocardiographic parameters such as LV end-diastolic dimension, LV mass, and ejection fraction, reflecting variability in training duration, athlete populations, and imaging timing relative to exercise. In contrast, HIIT studies showed generally lower heterogeneity (I^2^ ≈ 25–40%) for strain and torsional indices, suggesting more uniform responses to structured interval regimens. For acute exercise testing, I^2^ values were higher (often > 70%), particularly for myocardial strain and twisting–untwisting rates, consistent with the diverse nature of acute stress protocols and variable recovery measurement windows.

Across modalities, heterogeneity was lowest for heart rate and blood pressure responses (I^2^ < 30%), indicating broadly consistent hemodynamic adaptations, and highest for deformation metrics, where measurement technique, software vendor, and population training status likely contributed to dispersion. Importantly, substantial variability in speckle-tracking methodology—including differences in ultrasound vendor, frame rate, analysis software, segmentation algorithms, and timing of image acquisition—represents an additional major source of heterogeneity. These technical factors can themselves introduce systematic differences of approximately 1–2% in GLS and related strain indices, independent of true biological variation.

Despite this variability, pooled effect directions were coherent—endurance training tended to reduce deformation indices transiently, HIIT enhanced contractile measures, and acute tests revealed marked but reversible perturbations. Overall, the I^2^ patterns indicate that while effect magnitudes varied, the qualitative trends across exercise types were robust, supporting the interpretability of pooled findings.

### 3.9. NIH Quality Assessment of Included Studies

Methodological quality was generally high across all studies. Most investigations achieved scores between 8 and 10, reflecting robust design and consistent reporting (Supplementary Materials S3 [20,21,22,23,24,25,26,27,28,29,30,31,32,33,34,35,36,37,38,39,40,41,42]). Endurance and HIIT studies demonstrated comparable quality profiles, while Acute test studies maintained satisfactory standards despite smaller sample sizes and observational designs.

### 3.10. Publication Bias Assessment

**Figure 4:**
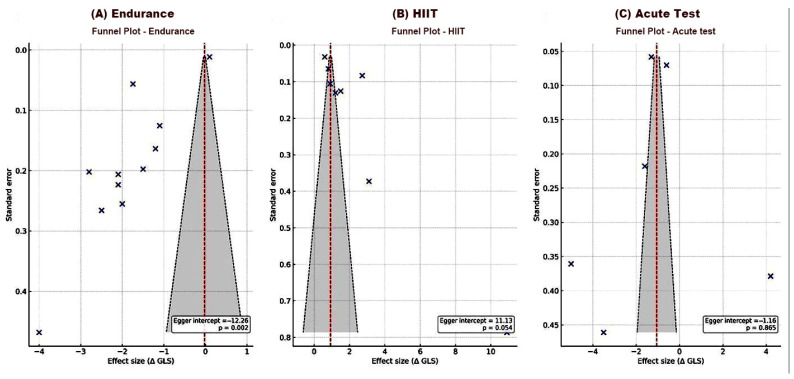
Funnel plots for publication bias assessment across exercise modalities. Funnel plots depicting the distribution of study effect sizes (Δ GLS) against their standard errors for (**A**) endurance training, (**B**) high-intensity interval training, and (**C**) acute exercise tests. The red dashed line represents the pooled mean effect, and the shaded area indicates the 95% confidence limits. GLS, global longitudinal strain; HIIT, high-intensity interval training.

As shown in Figure 4, visual inspection of the funnel plots did not suggest substantial asymmetry for most exercise modalities.

The distribution of studies appeared symmetrical for acute exercise tests, indicating a low likelihood of publication bias (Egger’s intercept = −1.16, *p* = 0.865). In contrast, the endurance training group demonstrated marked asymmetry, consistent with the presence of small-study effects (Egger’s intercept = −12.26, *p* = 0.002). The HIIT group showed a borderline pattern (Egger’s intercept = 11.13, *p* = 0.054), suggesting a possible but not definitive asymmetry.

The certainty of pooled estimates therefore varies by modality. For endurance training, certainty is lower due to the evident small-study effects and modest sample sizes. For HIIT, certainty can be considered moderate, as the borderline Egger’s result and relatively narrow study dispersion imply only partial bias risk. For acute test data, certainty remains limited by small sample numbers and heterogeneous protocols despite the absence of evident asymmetry.

In summary, the modality-wise certainty of evidence can be qualitatively graded as: low for endurance training, moderate for HIIT, and low-to-moderate for acute testing. These ratings highlight that publication bias and study heterogeneity may have influenced pooled magnitudes, and interpretation should prioritize overall directional trends rather than precise quantitative estimates.

## 4. Discussion

### 4.1. Summary of Main Findings

This systematic review synthesized findings from twenty-three studies published between 2009 and 2024 that evaluated the effects of endurance training, HIIT, and acute exercise tests on left ventricular (LV) mechanics assessed by speckle-tracking echocardiography. Together, these studies encompassed 859 participants, mainly healthy adults aged 23–35 years, and revealed distinct modality-specific adaptations in myocardial strain and torsional behavior. Importantly, our synthesis distinguishes between the acute effects of single prolonged endurance bouts and the chronic adaptations resulting from sustained endurance training. Overall, acute endurance exposure was characterized by transient and fully reversible reductions in global longitudinal, circumferential, and radial strains with concomitant decreases in torsion, whereas chronic endurance training maintained or modestly improved deformation indices without evidence of maladaptive remodeling. HIIT consistently produced significant improvements in deformation indices and rotational mechanics, and acute exercise protocols elicited short-lived, load-dependent fluctuations without lasting impairment. These collective results confirm that the direction and magnitude of LV mechanical adaptation depend primarily on the nature and duration of the exercise stimulus.

Acute endurance events produced a characteristic pattern of temporary LV fatigue accompanied by reversible mechanical alterations. Most studies conducted before and after marathon or ultra-endurance events demonstrated short-term reductions in global longitudinal, circumferential, and radial strains, together with impaired torsion and delayed untwisting—findings first described in triathletes and ultramarathon runners [20,21,22,23] and later confirmed in marathoners and cyclists [22,25,26]. These changes normalized within hours to days, consistent with transient systolic depression due to volume overload and post-exercise preload reduction rather than persistent dysfunction. In shorter-duration races or younger participants, the magnitude of these changes was smaller and recovery occurred within 24–48 h [24,30]. In contrast, chronic endurance training programs maintained normal strain values but increased basal rotation and altered strain–volume loop configuration [28], indicating physiological remodeling without impairment. In addition, mild post-race biomarker elevations (cTn, NT-proBNP) mirrored transient mechanical stress, reinforcing that these adaptations represent physiological fatigue, not pathological injury. Overall, endurance training promotes eccentric but functional remodeling and a limited capacity for additional contractile recruitment under acute stress rather than chronic dysfunction.

By contrast, HIIT consistently enhanced LV mechanics across diverse populations [31,32,33,34,35,36,37]. Short-term interventions of two to six weeks improved GLS, GCS, and torsion [33,34,35], and even single high-intensity bouts elicited acute strain increases [36]. Longer programs sustained these benefits without adverse remodeling. Improvements were evident across age and sex groups, including older men (improved GLS, blood pressure, and HR reserve) [34], women (enhanced systolic and diastolic indices) [32], and adolescents (increased subendocardial strain) [37]. Competitive athletes completing 18 weeks of HIIT maintained higher GLS with preserved torsion [31]. Quantitatively, HIIT increased GLS by approximately +2.7%, GCS by +3.3%, and GRS by +8.0%, accompanied by greater twisting (+23°/s) and untwisting (+22°/s) velocities, indicating augmented systolic efficiency and diastolic recoil.

Beyond left ventricular mechanics, several studies also reported modality-specific changes in RV and LA structure and function. Endurance and HIIT participants showed larger LA volumes and enhanced reservoir function compared with acute test groups, consistent with greater preload exposure and diastolic compliance. Post-exercise reductions in LA volume among endurance athletes and small, reversible increases after HIIT or acute tests suggest physiological, load-dependent remodeling rather than pathological dilation. Similarly, RV indices demonstrated adaptive patterns: endurance athletes exhibited higher baseline TAPSE and fractional area change with mild post-stress declines, while HIIT produced smaller but directionally similar effects. These biventricular and biatrial adaptations support increased stroke volume and venous return during sustained workloads while remaining fully reversible during recovery. Importantly, transient LA and RV strain reductions observed after endurance events may reflect mechanical fatigue and contribute to temporary alterations in atrial electrophysiology, providing a plausible functional substrate for benign, exercise-related atrial arrhythmias occasionally observed in endurance athletes.

Acute physiological tests demonstrated immediate, load-dependent responses reflecting transient myocardial stress. Isometric handgrip maneuvers increased apical strain and contractility [38], whereas maximal dynamic efforts induced short-term GLS depression consistent with exercise-induced fatigue [39]. In athletes without structural remodeling, strain values remained stable [40], while posture-related preload reduction lowered GLS and myocardial work indices [41]. Cardiopulmonary exercise testing confirmed that myocardial work, rather than GLS alone, best correlated with exercise capacity [42]. These findings collectively suggest that acute stress evokes rapid but reversible mechanical adjustments determined by the type and intensity of the applied load.

Across modalities, acute endurance events produced the largest transient reductions in myocardial strain, chronic endurance training maintained mechanical efficiency with stable deformation, HIIT elicited consistent functional enhancement, and acute laboratory stress induced reversible hemodynamic responses. RV and LA findings further support the concept of integrated biventricular and biatrial remodeling in response to exercise, characterized by functional, reversible dilation rather than permanent structural change. Between-study heterogeneity was moderate to high for endurance trials, lower for HIIT, and greatest for acute tests; nevertheless, the direction of adaptations was coherent—acute endurance transiently reduced deformation, HIIT enhanced it, and chronic endurance training preserved it. Small-study effects also varied by modality, being most apparent in endurance studies, borderline for HIIT, and limited for acute tests due to small sample sizes.

### 4.2. Comparison with Literature Data

The findings of this systematic review align with and extend existing evidence on exercise-induced cardiac remodeling and myocardial mechanics. The observation that endurance events are frequently associated with transient reductions in LV strain is consistent with earlier reviews and meta-analyses reporting exercise-induced cardiac fatigue. For example, Oxborough D. et al. [43] highlighted that prolonged strenuous exercise can depress both systolic and diastolic mechanics despite preserved ejection fraction, supporting our pooled results. Recent meta-analytic work [44] suggests modest acute reductions in GLS following prolonged endurance exercise, on the order of ~1%, closely mirroring the reductions observed in our endurance cohort. Collectively, these studies and our synthesis reinforce that such post-race changes are short-lived, physiological phenomena that resolve with recovery, rather than indicators of chronic maladaptive remodeling.

Conversely, our review demonstrated consistent improvements in LV deformation following HIIT, which is in line with the broader literature on interval training. Weston et al. [45] demonstrated superior gains in cardiorespiratory fitness and vascular health with HIIT compared to moderate continuous training. Similarly, Kemi O.J. et al. [46] showed that HIIT induces beneficial structural and functional adaptations at both cellular and whole-heart levels. More recently, McGregor et al. [47] confirmed in a large multicentre randomized controlled trial that HIIT is a safe, feasible, and highly effective training modality within cardiac rehabilitation, producing significant improvements in exercise capacity and cardiovascular outcomes in patients with coronary artery disease. In terms of strain imaging, our findings are corroborated by recent systematic reviews and meta-analyses [48,49,50] who reported significant increases in GLS after HIIT in various study groups. However, it should be emphasized that most studies included in our synthesis involved healthy or low-risk individuals, with relatively short intervention durations and small sample sizes. Therefore, safety statements regarding HIIT apply specifically to these populations. Extrapolations to patients with cardiovascular, valvular, or structural heart disease should be regarded as hypothesis-generating rather than evidence-based. These consistencies strengthen the interpretation that HIIT promotes physiological improvements in myocardial mechanics but warrant further confirmation in higher-risk clinical populations.

The heterogeneous responses observed in acute physiological tests are also reflected in the literature. For instance, Bhella P.S. et al. [51] demonstrated that posture and loading can strongly influence LV filling and mechanics, consistent with the positional variability reported in our included studies. Moreover, studies on short-term stress tests indicate that the direction of strain change is largely stimulus-dependent. La Gerche A. et al. [52] argued that while acute bouts may transiently impair mechanics, they also provide insight into myocardial reserve and adaptation.

Our results also resonate with sex- and age-specific observations. The improvements in strain with HIIT among women in our review are consistent with Andersen L.J. et al. [53] who reported favorable changes in diastolic function and strain parameters following structured aerobic programs. Similarly, a benefit in elderly patients was highlighted by the findings of Wisløff U. et al. [54], demonstrating HIIT’s broad applicability.

Nonetheless, the available studies remain short-term and lack hard clinical endpoints; thus, the current evidence does not yet support modifications in long-term management or perioperative strategies for higher-risk cardiac or surgical patients.

In summary, the collective literature supports the interpretation that transient post-race strain depression represents short-term fatigue, not chronic dysfunction, whereas HIIT and sustained endurance training evoke adaptive remodeling. At the same time, claims regarding the safety or generalizability of HIIT should remain restricted to the populations actually studied, and future research should explore its translational value in clinical and higher-risk cohorts.

### 4.3. Clinical Implications

The divergent patterns of LV mechanical adaptation seen across endurance, HIIT, and acute exercise protocols carry meaningful implications for sports cardiology, clinical echocardiography, and exercise prescription. The consistent observation that prolonged endurance exercise is followed by transient and reversible reductions in LV deformation—despite preserved ejection fraction—supports the concept of exercise-induced cardiac fatigue [55]. These temporary strain changes should not be interpreted as evidence of chronic maladaptation. In healthy athletes, these alterations in GLS, GCS, GRS, and torsion appear to be reversible, often normalizing within hours to days. Accordingly, transient post-event reductions in GLS or torsion observed immediately after extreme endurance efforts should not be overinterpreted as pathological, as they typically reflect physiological fatigue and acute load effects rather than intrinsic myocardial dysfunction. Echocardiographic evaluation performed during recovery, once hemodynamic stabilization has occurred, provides a more reliable assessment of baseline myocardial performance. Resting or adequately recovered measurements should therefore be preferred for athletic screening and preoperative decision-making.

However, the presence of strain abnormalities immediately after competition raises important considerations. For most athletes, these changes likely represent physiological fatigue, but in individuals with underlying susceptibility they could unmask latent dysfunction. Clinicians should therefore interpret acute post-race echocardiograms with caution, ensuring that abnormal strain findings are contextualized in relation to timing, load, and training background.

On the other hand, HIIT interventions demonstrated a consistent and reproducible pattern of improving LV mechanics, with nearly all studies showing significant gains in GLS and, in many cases, torsion and untwisting velocities [33,34,35]. Importantly, these changes occurred across diverse populations, including older sedentary men, middle-aged women, and adolescents, and were evident after both short-term and longer interventions. These findings reinforce that HIIT is a safe and physiologically adaptive training modality in both athletes and patients with cardiovascular disease [46,56]. Nonetheless, the underlying studies were small, short in duration, and largely conducted in healthy or low-risk participants. Accordingly, while HIIT appears effective and well tolerated within these populations, extrapolation to individuals with structural, valvular, or ischemic heart disease should be regarded as hypothesis-generating rather than definitive. Within its studied context, HIIT represents a time-efficient and physiologically sound strategy to enhance myocardial performance, and improvements in strain parameters often precede changes in ejection fraction, underscoring the value of deformation imaging as a sensitive marker of adaptive remodeling.

The acute test studies underscore the sensitivity of strain imaging to transient hemodynamic shifts and loading conditions. Isometric handgrip, brief maximal swimming bouts, or upright posture produced heterogeneous responses in LV mechanics, with some protocols eliciting improvements in regional strain while others induced global reductions [38,39,40,41,42]. These findings emphasize the need for standardized acquisition protocols during stress testing, as positional or loading differences can substantially influence results. Clinicians should therefore interpret strain responses during acute testing in the context of the applied stressor and the athlete’s underlying phenotype, rather than as absolute indicators of dysfunction.

Together, these findings carry several practical implications. First, acute post-endurance reductions in strain should be viewed as physiological and reversible, not maladaptive. Second, myocardial strain imaging provides greater sensitivity than ejection fraction for detecting subtle exercise-induced adaptations, and should be incorporated into athlete screening and longitudinal monitoring. Third, the consistent benefits of HIIT reinforce its prescription not only for athletic conditioning but also as a time-efficient strategy in preventive cardiology and cardiac rehabilitation. Finally, and importantly, exercise-modality–specific strain or torsion patterns should not currently be used in isolation to modify surgical or interventional indications. Rather, they may serve as complementary information in complex or borderline cases, helping to refine clinical judgment alongside standard morphological and functional parameters. Acute testing highlights the importance of contextual interpretation, with deformation imaging offering unique insights into myocardial reserve but requiring careful methodological standardization.

For clinicians, recognizing these modality-specific signatures is critical to differentiating physiological adaptation from pathology, tailoring exercise recommendations, and advancing the safe integration of exercise into cardiovascular health strategies.

Moreover, when interpreting deformation abnormalities in athletes, the potential presence of an underlying cardiomyopathic substrate—particularly in genotype-positive but phenotype-negative individuals—should be carefully considered. Subclinical or early-stage cardiomyopathies may initially present with normal morphology yet subtle impairments in strain or torsional mechanics, potentially confounding the distinction between physiological adaptation and early disease expression. The widespread use of genetic testing has increased the identification of such individuals, creating a diagnostic dilemma where sport participation itself may influence phenotypic evolution. Environmental factors and sustained exercise loads could amplify the expression of pathogenic variants, thereby unmasking or accelerating myocardial remodeling [57,58]. Accordingly, careful baseline evaluation—including family history, ECG, and advanced imaging—is essential before attributing strain alterations solely to training effects, ensuring early recognition of occult cardiomyopathy and the safe management of genotype-positive/phenotype-negative athletes.

### 4.4. Limitations of the Included Studies

This review has several important limitations. Most endurance data derive from acute observational studies; therefore, conclusions primarily concern short-term physiological responses rather than chronic outcomes. While they provide valuable insight into transient LV adaptations, they cannot establish long-term effects or causality. HIIT studies, although generally prospective, differed in intervention duration, intensity, and participant characteristics, contributing to variability in outcomes. Similarly, acute test protocols varied in type and load, limiting comparability across studies.

Although all studies used speckle-tracking echocardiography, heterogeneity in vendor platforms, frame rates, and analysis algorithms may have influenced strain measurements. Moderate-to-high I^2^ values further reflect differences in study design, participant demographics, and particularly in the timing of post-exercise imaging, which ranged from immediately after exertion to several hours into recovery. Such temporal variability likely affected LV strain and torsional parameters and should be considered when interpreting pooled results. To enhance reproducibility and facilitate future meta-analyses, upcoming trials should systematically report intra- and inter-observer variability as well as detailed acquisition settings, including frame rates, vendor-specific software, and tracking parameters.

Sub-analyses adjusted for age, sex, and imaging timing were not feasible due to incomplete and inconsistent data reporting. Future studies should adopt standardized imaging protocols and enable stratified analyses to clarify these sources of heterogeneity.

Finally, the absence of large randomized controlled trials remains a major limitation. Most available evidence derives from small, non-randomized, or observational studies. Well-designed RCTs comparing endurance and HIIT interventions are needed to establish causality and confirm the reproducibility and safety of the observed LV mechanical adaptations. The accuracy of basal longitudinal strain (BLS) may also be affected by anatomical factors—such as anterior chest wall deformities or pectus excavatum—that can induce local compressive artifacts and alter regional tracking performance [59,60,61].

### 4.5. Future Directions

Future research should aim to overcome the methodological and conceptual gaps highlighted in this review. First, larger and more rigorously designed studies are needed, particularly randomized controlled trials (RCTs) of exercise interventions that include both endurance and HIIT modalities. Most available data stem from small, single-center studies with fewer than 30 participants, which limits statistical power and generalizability. Adequately powered multicenter RCTs would help clarify the true magnitude and clinical relevance of exercise-induced changes in LV mechanics. Second, standardization of imaging protocols is essential. The widespread use of STE provides sensitive markers of myocardial deformation, but variability across vendors, frame rates, and analysis methods reduces comparability. International consensus on acquisition and reporting standards, similar to efforts in cardiac magnetic resonance, would enhance reproducibility and enable meaningful pooling of data across studies. Reporting of intra- and inter-observer variability should also become routine to strengthen confidence in observed effects. Third, future investigations should include more diverse populations. The majority of included studies focused on male athletes, often leaving female participants, older adults, and individuals with cardiovascular risk factors underrepresented. Yet, emerging evidence suggests sex-specific differences in exercise adaptation and heightened relevance of HIIT in post-menopausal women. Inclusion of broader populations will improve external validity and help determine whether current findings extend to patient groups where exercise training is increasingly prescribed. Fourth, longitudinal studies should clarify whether repeated episodes of acute endurance-induced “myocardial fatigue” lead to cumulative effects or remain benign adaptations. Current evidence suggests reversibility, but long-term follow-up is needed to exclude maladaptive remodeling in extreme endurance athletes. Finally, integration of myocardial work indices, cardiac biomarkers, and advanced imaging such as cardiac magnetic resonance could complement strain analyses, offering a more comprehensive understanding of exercise-induced adaptations. A multimodality approach may also clarify the links between acute responses, chronic adaptation, and clinical outcomes.

## 5. Conclusions

This systematic review differentiates between acute and chronic endurance adaptations. Acute endurance exercise, particularly in ultra-endurance contexts, is consistently associated with transient, reversible reductions in myocardial strain and torsional mechanics, while chronic endurance training appears neutral or mildly adaptive without evidence of dysfunction. In contrast, HIIT reliably enhances strain and rotational indices across diverse populations, supporting its role in both athletic development and cardiovascular rehabilitation. Acute physiological stress tests reveal heterogeneous, load-dependent changes that underscore the sensitivity of strain imaging. Collectively, these findings clarify that short-term strain depression after endurance events reflects physiological fatigue rather than chronic maladaptive remodeling, emphasizing the importance of temporal context when interpreting cardiac deformation responses.

## Figures and Tables

**Table 1:** Summary of studies [20,21,22,23,24,25,26,27,28,29,30] investigating the acute and subacute effects of endurance exercise on biventricular mechanics using two-dimensional speckle-tracking echocardiography. ACS, apical circumferential strain; cTnT, cardiac troponin T; CRIT, endurance cycling race intervention; GCS, global circumferential strain; GE, General Electric; GLS, global longitudinal strain; GRS, global radial strain; LASr, left atrial reservoir strain; LV, left ventricle; NS, not specified; RASr, right atrial reservoir strain; RV, right ventricle.

Study Name, Publication Year and Country	Size (%Males)	Mean Age (Yrs)	Repeated Measures Assessment	Software	Endurance Exercise	Influence on Biventricular Mechanics
Nottin S. (2009) France [20]	23 (100)	40	within 3 days before and within 45 min of the race completion	GE	14 h triathlon race	Decreased LV longitudinal, circumferential and radial strains; slightly reduced/delayed twist; depressed/delayed untwisting
George K. (2009) U.K. [21]	19 (100)	41	24 h prior to the race and within 60 min of race finish	GE (>40 and <90 frames per second)	89-km Comrades Marathon	Post–race significant reduction in LV–GLS, LV–GCS and LV–GRS
Chan-Dewar F. (2010) U.K. [22]	14 (100)	32	24 h prior to the race and within 60 min of race completion	GE (between 40 and 80 frames per second)	42.2-km London marathon	Mild reduction in LV–GLS, significant reduction in ACS; not altered rotation and unchanged LV torsion
Oxborough D.L. (2011) U.K. [23]	16 (75)	42	24 h before starting the race, and within 1 h of race completion	GE (<90 frames/second)	161-km ultramarathon	Reduced LV strain in all planes (longitudinal, circumferential and radial); unchanged LV torsion; LASr impairment; RV dilatation with reduced RV strain.
Unnithan V.B. (2015) U.K. [24]	20 (100)	15.2	Prior to and 45–min post–race	GE	5 km cross–country race	Minor transient decrease in LV–GLS 45–min post–race.
Stewart G.M. (2015) Australia [25]	15 (100)	28	1.5 h before and after CRIT_60_	GE (NS)	60-min endurance cycling intervention (CRIT_60_)	Decreased LV–GLS and RV–GLS; unchanged LV torsion; increased cTnT.
Stewart G.M. (2017) Australia [26]	23 (100)	28	Before and after 90-min CRIT	GE (frame rate of 50–80 frames/sec)	90-min endurance cycling intervention	Transient reductions in LV–GLS, LV–GCS and RV–GLS
Sengupta S.P. (2018) India [27]	50 (88)	40.8	Before training and within 10 days of completion of marathon	GE (frame rate of 50–80 frames/sec)	42.2-km marathon	Decreased LV–GLS and LV–GCS; unchanged LV–GRS; increased NT–proBNP
Oxborough D.L. (2019) U.K. [28]	23 (100)	27.4	Baseline and after 24–wk training	GE (NS)	24-wk endurance training program	Unchanged LV–GLS; mild reduction in LV–GCS and LV–GRS; increased basal rotation
Pagourelias E.D. (2022) Greece [29]	27 (70.4)	45	24 h before starting the race, and within 10 min after finishing	GE (NS)	246 km ultra–marathon running race	Decreased LV–GLS and RV–GLS; unchanged LASr and RASr
Birat A. (2023) France [30]	20 (100)	15.8	Immediately before and 24-h after the race	GE (NS)	68.5-km competitive adventure race	Unchanged LV–GLS; reduced LV global myocardial work, LV twisting and untwisting

**Table 2:** Summary of studies [31,32,33,34,35,36,37] evaluating the effects of high-intensity interval training on left ventricular mechanics assessed by speckle-tracking echocardiography. ACS, apical circumferential strain; ARS, apical radial strain; BLS, basal longitudinal strain; BRS, basal radial strain; GCS, global circumferential strain; GE, General Electric; GLS, global longitudinal strain; GRS, global radial strain; HIIT, high-intensity interval training; LV, left ventricular; MLS, mid-longitudinal strain.

Study Name, Publication Year and Country	Size (%Males)	Mean Age (Yrs)	Repeated Measures Assessment	Software	HIIT Program	Influence on LV Mechanics
D’Ascenzi F. (2015) Italy [31]	91 (60%)	23.0	Before the competitive period and 18 ± 2 weeks later	GE (60–90 frames/s)	An 18-week, intensive training programme	Improved LV–GLS (↑BLS, MLS). Unchanged LV basal rotation, apical rotation, torsion, twisting and untwisting rate.
Egelund J. (2017) Denmark [32]	37 (0%)	53.5	Before and after the intervention period	GE	12-week period of high-intensity aerobic cycle training	Improved LV–GLS.
O’Driscoll J.M. (2018) U.K. [33]	40 (100%)	21.0	Baseline and postintervention measures	GE (frame rates > 60 Hz)	2-wk HIIT intervention	Improved LV–GLS. Improvement in LV apical rotation, ACS, ARS and LV torsion.
Grace F. (2018) Australia [34]	22 (100%)	62.7	Pre/post HIIT design	GE	6 weeks of HIIT	Improved LV–GLS.
Huang YC. (2019) Taiwan [35]	18 (100%)	21.4	Two days before pretraining and two days after post-training	Siemens	High-intensity interval training for 6 weeks	Improved LV–GLS, LV–GCS and LV–GRS. Increased LV peak basal rotation, peak apical rotation and LV torsion.
Edwards J.J. (2022) U.K. [36]	50 (50%)	22.9	Baseline and following HIIT in the recovery period	GE (frame rates > 60 Hz)	HIIT (three 30 s periods of maximal intensity cycling)	Improved LV–GLS, BRS and ACS. Increased apical rotation and LV torsion.
Kösemen D.S. (2024) Turkey [37]	19 (100%)	16.8	Pre/post HIIT design	GE	8-week HIIT program	Improved LV–GLS and LV–GCS.

**Table 3:** Summary of studies [38,39,40,41,42] investigating the acute effects of exercise testing on left ventricular mechanics assessed by speckle-tracking echocardiography. BCS, basal circumferential strain; CPET, cardiopulmonary exercise testing; GE, General Electric; GLS, global longitudinal strain; LS, longitudinal strain; LV, left ventricular; RV, right ventricular.

Study Name, Publication Year and Country	Size (%Males)	Mean Age (Yrs)	Repeated Measures Assessment	Software	Acute Test	Influence on LV Mechanics
Stefani L. (2009) Italy [38]	32 (100)	25.0	At rest and at the peak of effort	My Lab 30 echocardiograph/X–Strain software	Isometric stress test performed with a graduated handle dynamometer held in the dominant arm for 3 min	Improved LV–GLS and RV–GLS; significant increase in LV and RV mid-apical longitudinal strain
Liang C. (2017) China [39]	15 (60)	16.3	30 min before and immediately after the training program	GE (frame rate > 70 frames per second)	200 m high-intensity (increasing-load) exercise in swimming athletes (one-time intensive exercise)	Reduced LV–GLS, due to exercise–induced greater impairment in LS of basal and middle segments
Żebrowska A. (2019) Poland [40]	14 (100)	27.2	Before and immediately after stress test	GE	Incremental stress test on a cycling ergometer	Mild reduction in LV–GLS, BCS and basal rotation
Kandels J. (June 2023) Germany [41]	50 (100)	25.7	Athletes examined in left lateral position and with upright posture	GE	Upright posture	Upright posture produced a marked reduction in global LV deformation with predominant regional depression in basal inferior/posterolateral segments.
Kandels J. (October 2023) Germany [42]	19 (100)	22.1	At rest and 5 min after CPET	GE	Incremental CPET in football players	Mild reduction in LV–GLS; unchanged global myocardial work

**Table 4:** Comparison of demographic and hemodynamic parameters across studies investigating endurance exercise, high-intensity interval training, and acute exercise tests. Data are presented as study-level summaries (medians with ranges). BMI, body mass index; DBP, diastolic blood pressure; HIIT, high-intensity interval training; HR, heart rate; NR, not reported; SBP, systolic blood pressure.

Parameter	Endurance (*n* = 250)	HIIT (*n* = 277)	Acute Tests (*n* = 130)	*p*-Value
**%males**	93.9 (70.4, 100.0)	72.9 (0.0, 100.0)	92.0 (60.0, 100.0)	0.119
**Mean age (yrs)**	32.3 (15.2, 45.0)	31.6 (16.8, 62.7)	23.3 (16.3, 27.2)	0.296
**Resting BMI (Kg/m^2^)**	23.1 (20.2, 26.3)	24.4 (21.5, 29.4)	23.4 (22.4, 24.1)	0.850
**Post–stress BMI (Kg/m^2^)**	22.5 (19.9, 24.3)	23.9 (21.5, 29.1)	NR	0.602
**Delta BMI (Kg/m^2^)**	−0.5 (−1.3, 0.1)	−0.1 (−0.3, 0.3)	NR	0.093
**Resting HR (bpm)**	61.1 (52.0, 74.1)	66.4 (62.2, 70.4)	67.5 (63.0, 79.1)	0.038
**Post-stress HR (bpm)**	73.9 (58.0, 86.6)	67.1 (57.7, 100.0)	121.3 (61.0, 185.0)	0.001
**Delta HR (bpm)**	12.8 (−9.6, 24.0)	0.7 (−5.5, 31.0)	53.9 (−18.1, 122.0)	0.005
**Rest SBP (mmHg)**	122.9 (118.0, 134.0)	120.9 (113.0, 135.0)	125.2 (118.6, 133.8)	0.508
**Post-stress SBP (mmHg)**	114.0 (107.0, 122.0)	115.7 (110.0, 130.0)	153.3 (125.3, 185.7)	<0.001
**Delta SBP (mmHg)**	−8.9 (−12.0, −3.0)	−5.2 (−15.0, 0.0)	26.4 (−3.0, 67.1)	<0.001
**Rest DBP (mmHg)**	73.8 (63.0, 82.0)	73.7 (67.6, 87.0)	73.8 (60.0, 82.2)	0.999
**Post-stress DBP (mmHg)**	70.3 (60.0, 79.0)	68.0 (59.0, 82.0)	70.3 (60.0, 78.0)	0.806
**Delta DBP** **(mmHg)**	−3.5 (–9.0, 0.0)	−5.6 (−10.0, −2.8)	–8.1 (−18.6, −1.4)	0.267

**Table 5:** Comparison of echocardiographic parameters at rest and post-stress among studies evaluating endurance exercise, high-intensity interval training, and acute exercise tests. Data are presented as study-level summaries (medians with ranges). HIIT, high-intensity interval training; IVS, interventricular septum; LAV, left atrial volume; LVEDD, left ventricular end-diastolic diameter; LVEDV, left ventricular end-diastolic volume; LVEF, left ventricular ejection fraction; LVESV, left ventricular end-systolic volume; RV-FAC, right ventricular fractional area change; NR, not reported; RVIT, right ventricular inflow tract; sPAP, systolic pulmonary artery pressure; SV, stroke volume; TAPSE, tricuspid annular plane systolic excursion.

Parameter	Endurance (*n* = 250)	HIIT (*n* = 277)	Acute Tests (*n* = 130)	*p*-Value
**Resting IVS (mm)**	9.5 (9.0, 11.4)	9.1 (7.4, 10.9)	9.9 (9.6, 10.2)	0.331
**Post-stress IVS (mm)**	10.7 (10.0, 11.4)	9.7 (7.4, 11.7)	10.2 (9.7, 10.8)	0.303
**Delta IVS (mm)**	0.8 (0.0, 1.4)	0.5 (0.0, 1.0)	0.4 (−0.2, 1.0)	0.388
**Resting LVEDD (mm)**	51.5 (47.0, 55.5)	47.1 (42.4, 51.0)	51.6 (48.2, 57.5)	0.021
**Post-stress LVEDD (mm)**	48.8 (46.0, 52.1)	47.7 (43.4, 52.0)	54.1 (53.4, 54.8)	0.008
**Delta LVEDD (mm)**	−1.4 (−3.4, −0.7)	0.6 (−1.7, 2.1)	1.2 (−4.1, 6.6)	0.174
**Resting LV mass (g)**	142.3 (120.2, 157.8)	160.0 (109.0, 223.0)	212.7 (206.4, 219.0)	0.011
**Post-stress LV mass (g)**	168.3 (160.3, 179.6)	170.5 (94.3, 241.0)	221.8 (221.0, 222.6)	0.079
**Delta LV mass (g)**	26.0 (16.0, 40.1)	10.5 (−14.7, 22.5)	9.1 (2.0, 16.2)	0.087
**Resting LVEDV (mL)**	126.8 (61.4, 169.0)	101.5 (93.3, 115.8)	140.9 (117.0, 164.8)	0.197
**Post-stress LVEDV (mL)**	117.4 (72.8, 162.0)	99.7 (85.3, 116.2)	148.2 (138.6, 157.9)	0.040
**Delta LVEDV (mL)**	−5.1 (−20.0, 11.4)	−1.8 (−8, 2.2)	7.3 (−26.2, 40.9)	0.59
**Resting LVESV (mL)**	48.0 (21.9, 66.0)	40.3 (40.2, 40.3)	52.9 (48.3, 57.5)	0.338
**Post-stress LVESV (mL)**	46.9 (20.3, 61.0)	37.6 (32.3, 41.8)	56.4 (50.0, 62.7)	0.118
**Delta LVESV (mL)**	−1.1 (−5.0, 3.0)	−2.7 (−8.0, 1.5)	3.5 (−7.5, 14.4)	0.390
**Resting LVEF (%)**	64.8 (56.0, 75.1)	60.0 (55.6, 65.1)	61.7 (59.7, 65.8)	0.096
**Post-stress LVEF (%)**	63.6 (54.0, 76.8)	61.1 (53.5, 67.7)	62.7 (61.1, 64.3)	0.666
**Delta LVEF (%)**	−1.1 (−9.0, 7.3)	1.2 (−2.1, 5.0)	−0.1 (−1.5, 1.4)	0.499
**Resting SV (mL)**	88.7 (68.7, 100.0)	65.8 (55.1, 75.6)	90.3 (86.0, 94.7)	<0.001
**Post-stress SV (mL)**	80.4 (66.9, 95.0)	75.9 (55.8, 103.8)	65.3 (65.3, 65.3)	0.367
**Delta SV (mL)**	−8.4 (−17.0, −1.8)	10.1 (0.7, 38.1)	−29.4 (−29.4, −29.4)	<0.001
**Resting E/A**	1.9 (1.4, 2.8)	1.6 (1.1, 1.9)	1.8 (1.8, 1.9)	0.307
**Post-stress E/A**	1.5 (1.1, 2.3)	1.6 (1.2, 2.0)	1.2 (1.2, 1.2)	0.369
**Delta E/A**	−0.4 (−0.8, 0.2)	0.0 (−0.7, 0.2)	−0.6 (−0.6, −0.6)	0.005
**Resting E/e’**	6.8 (5.1, 9.0)	5.6 (4.4, 7.0)	4.9 (4.9, 4.9)	0.135
**Post-stress E/e’**	6.6 (4.6, 8.3)	5.5 (4.1, 7.5)	5.4 (5.4, 5.4)	0.267
**Delta E/e’**	−0.2 (−0.7, 0.5)	−0.1 (−0.4, 0.5)	0.5 (0.5, 0.5)	0.114
**Resting LAV (mL)**	46.9 (22.8, 62.0)	49.9 (48.5, 51.2)	7.7 (7.7, 7.7)	0.017
**Post-stress LAV (mL)**	42.0 (19.0, 57.0)	60.4 (55.2, 65.6)	18.2 (18.2, 18.2)	0.024
**Delta LAV (mL)**	−4.9 (−6.0, −3.8)	10.6 (6.7, 14.4)	10.5 (10.5, 10.5)	<0.001
**Resting RVIT (mm)**	41.5 (41.0, 42.0)	NR	NR	/
**Post-stress RVIT (mm)**	44.0 (43.0, 45.0)	NR	NR	/
**Delta RVIT (mm)**	2.5 (2.0, 3.0)	NR	NR	/
**Resting TAPSE (mm)**	27.0 (25.0, 29.0)	24.1 (23.1, 25.0)	19.0 (19.0, 19.0)	0.004
**Post-stress TAPSE (mm)**	24.0 (22.0, 26.0)	23.8 (22.7, 25.0)	20.0 (20.0, 20.0)	0.056
**Delta TAPSE (mm)**	−3.0 (−3.0, −3.0)	−0.2 (−0.4, 0.0)	1.0 (1.0, 1.0)	<0.001
**Resting RV-FAC (%)**	47.3 (43.0, 50.0)	NR	NR	/
**Post-stress RV-FAC (%)**	42.3 (38.0, 46.0)	NR	NR	/
**Delta RV-FAC (%)**	−5.0 (−7.0, −3.0)	NR	NR	/
**Resting sPAP (mmHg)**	28.0 (28.0, 28.0)	NR	23.7 (23.7, 23.7)	<0.001
**Post-stress sPAP (mmHg)**	25.0 (25.0, 25.0)	NR	22.5 (22.5, 22.5)	<0.001
**Delta sPAP (mmHg)**	−3.0 (−3.0, −3.0)	NR	−1.2 (−1.2, −1.2)	<0.001

**Table 6:** Comparison of myocardial deformation parameters at rest and post-stress among studies assessing endurance exercise, high-intensity interval training, and acute exercise tests. Data are presented as study-level summaries (medians with ranges). ALS, apical longitudinal strain; BCS, basal circumferential strain; BLS, basal longitudinal strain; BRS, basal radial strain; GCS, global circumferential strain; GLS, global longitudinal strain; GRS, global radial strain; HIIT, high-intensity interval training; LASr, left atrial reservoir strain; LV, left ventricle; MACS, mid-apical circumferential strain; MARS, mid-apical radial strain; MLS, mid-ventricular longitudinal strain; NR, not reported; RASr, right atrial reservoir strain; RV, right ventricle.

Parameter	Endurance (*n* = 250)	HIIT (*n* = 277)	Acute Tests (*n* = 130)	*p*-Value
**Resting LV–GLS (%)**	19.2 (16.1, 21.8)	17.6 (12.1, 20.2)	18.4 (17.1, 19.4)	0.239
**Post-stress LV–GLS (%)**	17.5 (14.0, 20.5)	20.3 (13.3, 29.2)	17.1 (13.5, 21.3)	0.103
**Delta LV–GLS (%)**	−1.7 (−4.0, 0.1)	2.7 (0.6, 10.9)	−1.3 (−5.0, 4.2)	0.002
**Resting LV–BLS (%)**	18.5 (16.6, 20.1)	18.7 (17.3, 20.1)	17.1 (16.0, 18.2)	0.329
**Post-stress LV–BLS (%)**	16.6 (15.5, 18.7)	19.4 (18.2, 20.6)	15.1 (11.6, 18.5)	0.110
**Delta LV–BLS (%)**	−1.9 (−3.3, −1.0)	0.7 (0.5, 0.9)	−2.0 (−6.6, 2.5)	0.363
**Resting LV–MLS (%)**	20.1 (19.9, 20.4)	20.2 (20.2, 20.2)	18.6 (18.1, 19.2)	0.002
**Post-stress LV–MLS (%)**	18.5 (17.6, 19.3)	20.8 (20.6, 20.9)	19.2 (14.3, 24.1)	0.630
**Delta LV–MLS (%)**	−1.7 (−2.3, −1.1)	0.6 (0.4, 0.7)	0.6 (−4.9, 6.0)	0.627
**Resting LV–ALS (%)**	25.0 (24.6, 25.8)	22.2 (20.3, 24.0)	19.0 (18.1, 19.8)	<0.001
**Post-stress LV–ALS (%)**	24.6 (23.4, 26.6)	22.7 (21.0, 24.3)	22.4 (20.7, 24.1)	0.203
**Delta LV–ALS (%)**	−0.5 (−2.1, 1.9)	0.5 (0.3, 0.7)	3.4 (0.9, 6.0)	0.059
**Resting LV–GCS (%)**	20.6 (17.2, 24.0)	15.2 (14.2, 16.2)	15.9 (15.9, 15.9)	0.007
**Post-stress LV–GCS (%)**	18.0 (14.7, 22.2)	18.4 (17.8, 19.1)	15.2 (15.2, 15.2)	0.326
**Delta LV–GCS (%)**	−2.6 (−4.5, −1.0)	3.3 (1.6, 4.9)	−0.7 (−0.7, −0.7)	<0.001
**Resting LV–BCS (%)**	21.2 (16.7, 25.0)	18.3 (14.4, 23.2)	15.9 (15.9, 15.9)	0.218
**Post-stress LV–BCS (%)**	19.4 (13.5, 25.0)	20.4 (17.0, 24.7)	15.2 (15.2, 15.2)	0.320
**Delta LV–BCS (%)**	−1.8 (−3.2, 0.0)	2.1 (1.5, 2.6)	−0.7 (−0.7, −0.7)	0.005
**Resting LV–MACS (%)**	24.0 (18.3, 26.9)	18.3 (14.9, 21.8)	NR	0.110
**Post-stress LV–MACS (%)**	21.5 (15.9, 25.0)	22.3 (18.2, 26.4)	NR	0.814
**Delta LV–MACS (%)**	−2.4 (−3.2, −1.7)	3.9 (3.3, 4.6)	NR	<0.001
**Resting LV–GRS (%)**	46.3 (31.9, 53.4)	26.0 (26.0, 26.0)	NR	0.010
**Post-stress LV–GRS (%)**	39.6 (30.8, 47.0)	34.0 (34.0, 34.0)	NR	0.292
**Delta LV–GRS (%)**	−6.7 (−13.1, −1.0)	8.0 (8.0, 8.0)	NR	0.004
**Resting LV–BRS (%)**	42.0 (36.3, 47.8)	39.3 (39.3, 39.3)	NR	0.567
**Post-stress LV–BRS (%)**	41.0 (30.6, 51.3)	23.2 (23.2, 23.2)	NR	0.105
**Delta LV–BRS (%)**	−1.1 (−5.7, 3.5)	−16.1 (−16.1, 16.1)	NR	0.022
**Resting LV–MARS (%)**	42.0 (39.4, 44.6)	35.5 (35.5, 35.5)	NR	0.044
**Post-stress LV–MARS (%)**	33.9 (31.1, 36.7)	47.5 (47.5, 47.5)	NR	0.007
**Delta LV–MARS (%)**	−8.1 (−13.5, −2.7)	12.0 (12.0, 12.0)	NR	0.015
**Resting RV–GLS (%)**	26.7 (22.9, 28.4)	NR	24.4 (24.4, 24.4)	0.235
**Post-stress RV–GLS (%)**	23.1 (21.2, 24.0)	NR	25.4 (25.4, 25.4)	0.034
**Delta RV–GLS (%)**	−3.6 (−4.9, −1.7)	NR	1.0 (1.0, 1.0)	0.006
**Resting LASr (%)**	42.0 (38.9, 45.2)	NR	NR	/
**Post-stress LASr (%)**	35.5 (34.0, 36.9)	NR	NR	/
**Delta LASr (%)**	−6.6 (−8.3, −4.9)	NR	NR	/
**Resting RASr (%)**	44.6 (44.6, 44.6)	NR	NR	/
**Post-stress RASr (%)**	44.3 (44.3, 44.3)	NR	NR	/
**Delta RASr (%)**	−0.3 (−0.3, −0.3)	NR	NR	/

**Table 7:** Comparison of left ventricular rotational mechanics parameters at rest and post-stress among studies evaluating endurance exercise, high-intensity interval training, and acute exercise tests. Data are presented as study-level summaries (medians with ranges). HIIT, high-intensity interval training; LV, left ventricle; NR, not reported.

Parameter	Endurance (*n* = 250)	HIIT (*n* = 277)	Acute Tests (*n* = 130)	*p*-Value
**Resting LV basal rotation (°)**	4.1 (2.2, 6.5)	5.0 (4.1, 5.6)	6.8 (6.8, 6.8)	0.045
**Post-stress LV basal rotation (°)**	4.6 (3.7, 5.7)	6.0 (5.2, 7.5)	6.1 (6.1, 6.1)	0.022
**Delta LV basal rotation (°)**	0.5 (−1.3, 2.3)	1.0 (0.3, 1.9)	−0.7 (−0.7, −0.7)	0.130
**Resting LV apical rotation (°)**	7.5 (3.7, 10.4)	5.7 (2.1, 7.9)	NR	0.250
**Post-stress LV apical rotation (°)**	6.2 (2.0, 9.4)	7.5 (4.0, 12.4)	NR	0.478
**Delta LV apical rotation (°)**	−1.3 (−2.6, 0.2)	1.8 (−1.1, 4.5)	NR	0.007
**Resting LV torsion (°)**	11.2 (6.7, 15.9)	9.2 (6.2, 10.6)	NR	0.277
**Post-stress LV torsion (°)**	9.7 (5.7, 15.1)	11.9 (9.0, 16.2)	NR	0.294
**Delta LV torsion (°)**	−1.4 (−3.9, 1.4)	2.8 (−0.3, 5.7)	NR	0.003
**Resting LV twisting (basal rotation rate) °/s**	59.9 (51.0, 68.4)	54.7 (48.3, 58.3)	NR	0.230
**Post-stress LV twisting (basal rotation rate) °/s**	47.7 (2.9, 67.7)	78.2 (59.4, 114.0)	NR	0.088
**Delta LV twisting (basal rotation rate) °/s**	−12.2 (−65.5, 13.4)	23.5 (1.8, 55.7)	NR	0.112
**Resting LV untwisting (basal rotation rate) °/s**	54.2 (27.7, 82.6)	53.9 (26.7, 94.4)	NR	0.980
**Post-stress LV untwisting (basal rotation rate) °/s**	46.1 (38.0, 54.3)	76.9 (46.3, 98.3)	NR	0.016
**Delta LV untwisting (basal rotation rate) °/s**	−8.1 (−28.3, 13.8)	23.0 (−2.4, 50.3)	NR	0.034
**Resting LV twisting (apical rotation rate) °/s**	63.4 (55.0, 69.6)	51.8 (45.8, 61.3)	NR	0.013
**Post-stress LV twisting (apical rotation rate) °/s**	61.9 (52.0, 76.4)	85.0 (61.0, 132.7)	NR	0.137
**Delta LV twisting (apical rotation rate) °/s**	−1.5 (−10.6, 11.1)	33.2 (12.9, 71.4)	NR	0.017
**Resting LV untwisting (apical rotation rate) °/s**	53.0 (29.0, 68.1)	60.2 (26.7, 94.4)	NR	0.612
**Post-stress LV untwisting (apical rotation rate) °/s**	47.1 (18.4, 71.6)	85.3 (59.8, 116.0)	NR	0.025
**Delta LV untwisting (apical rotation rate) °/s**	−5.8 (−10.6, 3.5)	25.1 (3.9, 41.5)	NR	0.004

## Data Availability

Data extracted from included studies will be publicly available on Zenodo (https://zenodo.org accessed on 6 October 2025).

## Supplementary Materials

The following supporting information can be downloaded at: https://www.mdpi.com/article/10.3390/jcm14228210/s1, Supplementary Materials S1: PRISMA 2020 checklist; Supplementary Materials S2: The full INPLASY record (https://inplasy.com/inplasy-2025-10-0002/ accessed on 23 October 2025); Supplementary Materials S3: NIH Quality Assessment of Included Studies [20,21,22,23,24,25,26,27,28,29,30,31,32,33,34,35,36,37,38,39,40,41,42].
