# Supplementary material for: Influence of Endurance Training, High-Intensity Interval Training, and Acute Exercise on Left Ventricular Mechanics: A Systematic Review
Source: J Clin Med. 2025 Nov 19;14(22):8210. doi: 10.3390/jcm14228210 (PMC12653759; doi:10.3390/jcm14228210)
Supplement: Supplementary file 1 [file jcm-14-08210-s001.zip › Supplementary Materials S2.pdf]

INPLASY

Influence of Endurance Training, High-Intensity Interval Training, and Acute Exercise on Left Ventricular Mechanics: A Systematic Review

INPLASY2025100002

doi: 10.37766/inplasy2025.10.0002

Received: 2 October 2025

Published: 2 October 2025

Sonaglioni, A; Nicolosi, GL; Baravelli, M.

Corresponding author:
Andrea Sonaglioni

sonaglioniandrea@gmail.com

Author Affiliation:
IRCCS MultiMedica.

ADMINISTRATIVE INFORMATION

Support - N/A.

Review Stage at time of this submission - The review has not yet started.

Conflicts of interest - None declared.

INPLASY registration number: INPLASY2025100002

Amendments - This protocol was registered with the International Platform of Registered Systematic Review and Meta-Analysis Protocols (INPLASY) on 2 October 2025 and was last updated on 2 October 2025.

INTRODUCTION

Review question / Objective In this review and meta-synthesis we examine studies that evaluate left ventricular (LV) mechanics—both conventional echocardiographic parameters and deformation metrics—across endurance training, high-intensity interval training (HIIT), and acute test protocols. By grouping studies into these three categories and comparing their reported changes in chamber size, mass, ejection fraction, as well as strain and torsional indices, our aim is to delineate the mechanical "signatures" of each training modality. We hypothesize that endurance stimuli will more frequently be associated with transient reductions in deformation, while HIIT will show consistent improvements, and acute tests will demonstrate variable but revealing changes in mechanical reserve.

Rationale In athletes or trained populations, acute and chronic exercise may exert distinct influences. Acute endurance exposure—such as marathon or ultramarathon events—may lead to transient "myocardial fatigue," with reductions in strain and delayed untwisting immediately after completion. Over chronic time frames, endurance training might lead to structural remodeling (eccentric hypertrophy) without detrimental impact on strain indices. Alternatively, HIIT imposes repeated stress–rest cycles and may drive more efficient contractile adaptations, possibly enhancing strain or torsional reserve. Meanwhile, controlled acute tests (e.g. isometric or short maximal efforts) serve as probes of myocardial reserve and stress responsiveness in the short term.

Despite the growing use of strain imaging in exercise physiology, direct comparisons between endurance, HIIT, and acute test models remain limited. Many existing studies focus on single modalities or single populations, making inferences across studies difficult. A systematic collation of

Sonaglioni et al. INPLASY protocol 2025100002. doi:10.37766/inplasy2025.10.0002 Downloaded from https://inplasy.com/inplasy-2025-10-0002/

evidence across these three paradigms may help clarify whether endurance-type stimuli inherently depress mechanics acutely, whether HIIT more reliably enhances deformation, and to what extent acute testing reveals latent mechanical reserve.

Condition being studied Regular physical exercise triggers profound adaptations in cardiac structure and function, which have been the subject of intense investigation for decades. Traditionally, studies of the "athlete's heart" have focused on gross parameters such as ventricular chamber size, wall thickness, and ejection fraction (EF). Yet, these conventional echocardiographic indices often remain within normal limits, even in the presence of subtle myocardial remodeling or dysfunction. In recent years, myocardial deformation imaging—particularly speckle-tracking echocardiography measuring strain, strain rate, and rotational mechanics—has emerged as a more sensitive tool to detect early or subclinical changes in LV performance.

Global longitudinal strain (GLS) in particular is known to decline before overt changes in EF occur, and is prognostically informative in cardiovascular disease settings. A recent meta-analysis examined how exercise interventions influence LV GLS and found that, while significant improvements were evident in populations with cardiovascular disease, in healthy or low-risk individuals the effect was more variable and often modest. This ambiguity highlights the need for more mechanistic insight into how different forms of exercise affect LV mechanics under diverse loading and adaptation conditions.

From a physiological perspective, LV mechanics encompass not only longitudinal deformation but also circumferential shortening, radial thickening, and twist–untwist (torsion). These components collectively contribute to efficient systolic ejection and diastolic recoil. Exercise imposes complex alternating demands on preload, afterload, and contractile force, and the adaptation of each mechanical component may depend on the nature, intensity, and duration of the stimulus. Earlier work on healthy individuals suggests that exercise training can induce regional heterogeneity in LV systolic function, possibly mediated by concomitant adaptations of the right ventricle and ventricular interdependence.

METHODS

Search strategy We will conduct a comprehensive literature search to identify studies evaluating the effects of different exercise training modalities on LV mechanics assessed by speckle-tracking echocardiography. The search will be performed in PubMed, Scopus, and EMBASE databases from inception through October 2025, with no language restrictions. The search strategy will combine Medical Subject Headings (MeSH) and free-text terms, including: "endurance training," "high-intensity interval training," "acute exercise," "left ventricular mechanics," "speckle-tracking," "strain," "torsion," and "echocardiography." Boolean operators ("AND," "OR") will be applied to maximize sensitivity. In addition, the reference lists of relevant systematic reviews and included articles will be screened manually to identify additional eligible studies.

Participant or population Studies will be considered eligible if they are original research articles published in peer-reviewed journals, evaluating the effects of physical training or acute exercise on left ventricular mechanics using speckle-tracking echocardiography. We will include studies that investigated healthy participants across a range of activity levels, from competitive athletes and recreationally active individuals to sedentary subjects undergoing structured training interventions. Eligible exercise exposures will comprise endurance training, high-intensity interval training, or acute physiological tests such as isometric exercise, short maximal efforts, or cardiopulmonary exercise testing. To be included, studies will be required to report at least one echocardiographic measure of LV deformation, such as global longitudinal strain (GLS), circumferential strain, radial strain, or torsional parameters, either before and after the intervention or between different training phases. Conventional echocardiographic indices and biomarkers will also be extracted when available.

Exclusion criteria will include case reports, conference abstracts without sufficient data, narrative reviews, and editorials. We will also exclude studies conducted in populations with overt cardiovascular disease, structural cardiac abnormalities, or other clinical conditions that might independently alter LV mechanics. When duplicate reports derived from the same cohort will be identified, the most complete or most recent study will be retained.

Intervention Mean differences (Δ) of main LV conventional and functional echocardiographic parameters between pre- and post-exercise or pre- and post-training values will be calculated. For pooled comparisons, Δ values will be normalized across studies and grouped by exercise modality (endurance, HIIT, acute tests).

Comparator Mean differences (Δ) of main LV conventional and functional echocardiographic

Sonaglioni et al. INPLASY protocol 2025100002. doi:10.37766/inplasy2025.10.0002 Downloaded from https://inplasy.com/inplasy-2025-10-0002/

parameters between pre- and post-exercise or pre- and post-training values will be calculated. For pooled comparisons, Δ values will be normalized across studies and grouped by exercise modality (endurance, HIIT, acute tests).

Study designs to be included Observational Cohort and Cross-Sectional Studies.

Eligibility criteria Studies will be considered eligible if they are original research articles published in peer-reviewed journals, evaluating the effects of physical training or acute exercise on left ventricular mechanics using speckle-tracking echocardiography. We will include studies that investigated healthy participants across a range of activity levels, from competitive athletes and recreationally active individuals to sedentary subjects undergoing structured training interventions. Eligible exercise exposures will comprise endurance training, high-intensity interval training, or acute physiological tests such as isometric exercise, short maximal efforts, or cardiopulmonary exercise testing. To be included, studies will be required to report at least one echocardiographic measure of LV deformation, such as global longitudinal strain (GLS), circumferential strain, radial strain, or torsional parameters, either before and after the intervention or between different training phases. Conventional echocardiographic indices and biomarkers will also be extracted when available.
Exclusion criteria will include case reports, conference abstracts without sufficient data, narrative reviews, and editorials. We will also exclude studies conducted in populations with overt cardiovascular disease, structural cardiac abnormalities, or other clinical conditions that might independently alter LV mechanics. When duplicate reports derived from the same cohort will be identified, the most complete or most recent study will be retained.

Information sources We will conduct a comprehensive literature search to identify studies evaluating the effects of different exercise training modalities on LV mechanics assessed by speckle-tracking echocardiography. The search will be performed in PubMed, Scopus, and EMBASE databases from inception through October 2025, with no language restrictions. The search strategy will combine Medical Subject Headings (MeSH) and free-text terms, including: “endurance training,” “high-intensity interval training,” “acute exercise,” “left ventricular mechanics,” “speckle-tracking,” “strain,” “torsion,” and “echocardiography.” Boolean operators (“AND,” “OR”) will be applied to maximize sensitivity. In addition, the reference lists of relevant systematic reviews and included articles will be screened manually to identify additional eligible studies.

Main outcome(s) To assess the effect of endurance training, HIIT, and acute test protocols on LV mechanics—both conventional echocardiographic parameters and deformation metrics.

Quality assessment / Risk of bias analysis The methodological quality of the included studies will be assessed using the National Institutes of Health (NIH) Quality Assessment Tool for Observational Cohort and Cross-Sectional Studies. This instrument evaluates 14 domains, including clarity of the research question, definition of the study population, participation rate, exposure and outcome measures, adequacy of statistical analyses, and length of follow-up for intervention studies. Each study will be independently rated by the three reviewers who will perform data extraction, and classified as good, fair, or poor quality according to NIH guidelines.

Strategy of data synthesis Two investigators will independently screen titles and abstracts retrieved from the initial search to exclude irrelevant records. Full-texts will then be assessed for eligibility. Discrepancies will be resolved by consensus with a third reviewer. Data extraction will be performed independently by three experienced cardiologists in October 2025, using a standardized form. Extracted data will include: study author, year of publication, country, sample size, age and sex distribution, population characteristics (athletes vs. non-athletes), exercise modality and duration, study design, echocardiographic platform, imaging parameters (GLS, GCS, GRS, torsion, twist/untwist rates), conventional echocardiographic indices, and biomarker data when available. Data will be cross-checked for accuracy and summarized in structured tables.

Subgroup analysis N/A.

Sensitivity analysis N/A.

Country(ies) involved Italy.

Keywords left ventricular mechanics; speckle-tracking echocardiography; endurance training; high-intensity interval training; acute exercise; strain; torsion.

Contributions of each author
Author 1 - Andrea Sonaglioni - Author 1 will draft the manuscript.

Sonaglioni et al. INPLASY protocol 2025100002. doi:10.37766/inplasy2025.10.0002 Downloaded from https://inplasy.com/inplasy-2025-10-0002/

Email: sonaglioniandrea@gmail.com
Author 2 - Gian Luigi Nicolosi - Author 2 will critically revise the manuscript.
Email: gianluigi.nicolosi@gmail.com
Author 3 - Massimo Baravelli - Author 3 will critically revise the manuscript.
Email: massimo.baravelli@multimedica.it

Sonaglioni et al. INPLASY protocol 2025100002. doi:10.37766/inplasy2025.10.0002 Downloaded from https://inplasy.com/inplasy-2025-10-0002/
