# Supplementary material for: Influence of Endurance Training, High-Intensity Interval Training, and Acute Exercise on Left Ventricular Mechanics: A Systematic Review
Source: J Clin Med. 2025 Nov 19;14(22):8210. doi: 10.3390/jcm14228210 (PMC12653759; doi:10.3390/jcm14228210)
Supplement: Supplementary file 1 [file jcm-14-08210-s001.zip › Supplementary Materials S3.pdf]

NIH Quality Assessment of Included Studies

Study name	Group	Q1	Q2	Q3	Q4	Q5	Q6	Q7	Q8	Q9	Q10	Q11	Q12	Q13	Q14	Quality (Total Quality Score)
Nottin S. (2009) France	Endurance	Yes	Yes	NR	Yes	NR	Yes	Yes	NR	Yes	Yes	No	Yes	No	No	8
George K. (2009) UK	Endurance	Yes	Yes	NR	Yes	No	Yes	Yes	NR	Yes	Yes	No	Yes	Yes	No	9
Chan-Dewar F. (2010) U.K.	Endurance	Yes	Yes	NR	Yes	No	Yes	Yes	NR	Yes	Yes	No	Yes	Yes	No	9
Oxborough D.L. (2011) UK	Endurance	Yes	Yes	NR	Yes	NR	Yes	Yes	NR	Yes	Yes	No	Yes	No	No	8
Unnithan V.B. (2015) UK	Endurance	Yes	Yes	No	Yes	No	Yes	NR	NR	Yes	Yes	No	Yes	Yes	No	8
Stewart G.M. (2015) Australia	Endurance	Yes	Yes	NR	Yes	No	Yes	Yes	NR	Yes	Yes	No	Yes	Yes	Yes	10
Stewart G.M. (2017) Australia	Endurance	Yes	Yes	NR	Yes	NR	Yes	Yes	NR	Yes	Yes	No	Yes	No	No	8
Sengupta S.P. (2018) India	Endurance	Yes	Yes	NR	Yes	No	Yes	Yes	NR	Yes	Yes	No	Yes	Yes	No	9
Oxborough D.L. (2019) UK	Endurance	Yes	Yes	No	Yes	No	Yes	NR	NR	Yes	Yes	No	Yes	Yes	No	8
Pagourelias E.D. (2022) Greece	Endurance	Yes	Yes	NR	Yes	NR	Yes	Yes	NR	Yes	Yes	No	Yes	No	No	8
Birat A. (2023) France	Endurance	Yes	Yes	NR	Yes	No	Yes	Yes	NR	Yes	Yes	No	Yes	Yes	Yes	10
D'Ascenzi F. (2015) Italy	HIIT	Yes	Yes	NR	Yes	NR	Yes	Yes	NR	Yes	Yes	No	Yes	No	No	8
Egelund J. (2017) Denmark	HIIT	Yes	Yes	NR	Yes	No	Yes	Yes	NR	Yes	Yes	No	Yes	Yes	No	9

O'Driscoll J.M. (2018) U.K.	HIIT	Yes	Yes	NR	Yes	No	Yes	Yes	NR	Yes	Yes	No	Yes	Yes	No	9
Grace F. (2018) Australia	HIIT	Yes	Yes	NR	Yes	NR	Yes	Yes	NR	Yes	Yes	No	Yes	No	No	8
Huang YC. (2019) Taiwan	HIIT	Yes	Yes	No	Yes	No	Yes	NR	NR	Yes	Yes	No	Yes	Yes	No	8
Edwards J.J. (2022) U.K.	HIIT	Yes	Yes	NR	Yes	No	Yes	Yes	NR	Yes	Yes	No	Yes	Yes	No	9
Kösemen D.S. (2024) Turkey	HIIT	Yes	Yes	NR	Yes	NR	Yes	Yes	NR	Yes	Yes	No	Yes	No	No	8
Stefani L. (2009) Italy	Acute tests	Yes	Yes	No	Yes	No	Yes	NR	NR	Yes	Yes	No	Yes	Yes	No	8
Liang C. (2017) China	Acute tests	Yes	Yes	NR	Yes	NR	Yes	Yes	NR	Yes	Yes	No	Yes	No	No	8
Żebrowska A. (2019) Poland	Acute tests	Yes	Yes	NR	Yes	No	Yes	Yes	NR	Yes	Yes	No	Yes	Yes	No	9
Kandels J. (Jun 2023) Germany	Acute tests	Yes	Yes	NR	Yes	No	Yes	Yes	NR	Yes	Yes	No	Yes	Yes	No	9
Kandels J. (Oct 2023) Germany	Acute tests	Yes	Yes	NR	Yes	NR	Yes	Yes	NR	Yes	Yes	No	Yes	No	No	8
